# Cervical lymph node metastasis in squamous cell carcinoma of the buccal mucosa: a retrospective study on pattern of involvement and clinical analysis

**DOI:** 10.4317/medoral.24016

**Published:** 2020-12-19

**Authors:** Nadimul Hoda, Rajani BC, Subhabrata Ghosh, Sabitha KS, Vasantha Dhara B, Jayesh Nathani

**Affiliations:** 1 Assistant professor, department of oral oncology, Kidwai Memorial Institue of Oncology, Bengaluru, Karnataka; 2 Fellow, department of oral oncology, Kidwai Memorial Institue of Oncology, Bengaluru, Karnataka; 3 Professor and head, department of oral oncology, Kidwai Memorial Institue of Oncology, Bengaluru, Karnataka

## Abstract

**Background:**

The study was performed with an aim to map the pattern of metastasis of squamous cell carcinomas of buccal mucosa to various cervical lymph node levels and analyze its correlation with primary tumor size and histo-pathological grading.

**Material and Methods:**

254 patients with squamous cell carcinoma of the buccal mucosa treated with surgery first approach were analyzed retrospectively. The tumor size was noted from pre-operative CT Scans and were divided into early and advanced tumors. The resected specimen was studied to note the histo-pathological grading of the squamous cell carcinoma and the metastatic deposits at various lymph node levels.

**Results:**

Out of 254 patients (149 females, 105 males), 145 patients showed histo-pathologically proven metastatic deposits in one or more lymph nodes out of which there were 56 patients showing occult metastasis. 78/145 patients showed metastatic involvement of level IB and/or IA lymph nodes, 31 showed involvement of level II and/or I lymph nodes, 27 showed involvement of level III with or without involvement of level I and II and 9 showed metastasis to level IV and V lymph nodes with or without level I, II or III lymph nodes. Cervical lymph node metastasis had statistically significant association with tumor size with advanced tumors showing worse pattern of metastatic spread beyond level I and II lymph nodes. As the degree of differentiation of squamous cell carcinoma reduced, they were more prone for cervical metastasis with moderately and poorly differentiated squamous cell carcinoma showing higher involvement of level III, IV and V lymph nodes.

**Conclusions:**

The majority of buccal mucosa cases showed metastasis to level I, II and III lymph nodes out of which level IB and/or IA was most frequently involved. Metastasis to level IV and V lymph nodes was rare and was seen especially in patients with advanced primary tumor and poor histo-pathologic differentiation.

** Key words:**Oral squamous cell carcinoma (OSCC), cervical lymph node metastasis, histologic differentiation, locally advanced disease.

## Introduction

One of the most common sites for cancer in the head and neck region is the oral cavity with squamous cell carcinoma being the most common type of malignancy (1,2). In oral cavity, buccal mucosa is the most common site of occurrence of carcinoma amongst the South Asian population (3). Oral squamous cell carcinomas (OSCC) carry with them a high tendency for cervical lymph node metastasis which is proven to be the most significant independent prognostic factor, reducing the survival rate of the patient by 50% (4). Thus, an optimum treatment of the cervical lymph nodes becomes absolutely necessary for better prognostic outcomes. Literature holds proof of the fact that surgical treatment, that is neck dissection is the best treatment protocol for cervical lymph node metastasis in OSCC (5). Although consensus is reached on the mode of treatment, there is considerable debate still persisting on the extent and magnitude of neck dissection. The main reason for this dilemma being the inability to detect the extent of lymph node involvement in patients with OSCC. In the present day besides clinical examination, there are various diagnostic aids to detect lymph node metastasis, like Computed Tomography Scans (CT Scans), Magnetic Resonance Imaging (MRI) and Ultrasonography (USG), but their efficacy in detecting cervical lymph node metastasis is still debated (5). This problem is further worsened by the presence of occult metastasis in these patients. It has been seen that as high as 25% of clinically negative necks show metastasis to cervical lymph nodes after neck dissection (6,7).

The purpose of this study was to evaluate the neck node metastasis pattern in cases of OSCC of buccal mucosa. The extent of occult metastasis and the involvement of various levels of lymph nodes were analyzed and they were correlated with the size of the primary tumor and the histo-pathological grading.

## Material and Methods

This was a retrospective study conducted in the Department of Oral Oncology in Kidwai Memorial Institute of Oncology, Bengaluru, India. Records of 254 patients who were treated for squamous cell carcinoma of the buccal mucosa from January 2017 till December 2019 were studied. As it is a retrospective study, the Institutional Review Board (IRB) waived off the ethical clearance and no informed consent had to be taken. The “clinical examination notes” of the case histories of all the patients were studied and pre-operative contrast enhanced CT Scans which were done less than one month before the date of surgery were referred to estimate the size of the primary tumor. Post-operative histopathology reports of the resected specimens were studied to note the histo-pathological grading and metastasis to various lymph node levels.

Patients who had a single lesion in the buccal mucosa which were histo-pathologically proven to be squamous cell carcinoma were included in this study. The patients who were treated by surgery first approach were chosen and all of them had undergone a Modified Radical Neck Dissection (MRND) or Selective Neck Dissection (SND) of level I, II, III, IV and V lymph nodes. Any patients who had pre-malignant conditions like oral submucous fibrosis or congenital syndromes like dyskeratosis congenita which predisposes to ocurrence of squamous cell carcinoma and conditions of generalized lymphadenopathy like tuberculosis, non-Hodgkin’s lymphoma etc. were excluded from the study.

The size of the primary was determined from the preoperative CT Scans. All lesions which were less than 4cm in its largest dimensions were categorized into early lesions (E) and the lesions which were more than 4cm in its largest dimensions were categorized as advanced lesions (A). Patients were divided into 2 groups, clinically neck positive (cN+) and clinically neck negative (cN0). The post-operative histopathology report of the resected neck dissection specimen was used for the pathologic grading of tumor according to World Health Organization (WHO) classification into well differentiated squamous cell carcinoma, moderately differentiated squamous cell carcinoma and poorly differentiated squamous cell carcinoma. The post-operative histo-pathological report was also used for staging of neck (pN) and the patients were divided into 2 groups pathological neck positive (pN+) and pathological neck negative (pN0). Amongst the pN+ patients, the involvement of levels of lymph nodes were noted (irrespective of the number of nodes involved) and it was correlated with the tumor stage (Early or advanced). Association between the histo-pathologic grading and cervical lymph node metastasis (pN+) and involvement of different levels of lymph nodes were tested.

In statistical analysis, chi –square test was done to check for correlation between levels of lymph node involvement and tumor size and Pearson correlation analysis was done to test any association between histo-pathological grading and lymph node involvement. Results were considered to be significant at a 5% critical level (*p*<0.05). Statistical analysis was done using IBM SPSS Statistics (ver. 22.0; IBM, Armonk, NY, USA).

## Results

A total of 254 patients who satisfied the inclusion and exclusion criteria were included in the study. 149 of them were females and 105 were males, and their ages ranged from 26 years to 74 years. 196/254 (77.17%) patients had any habit of chewing or smoking any form of tobacco and/or areca nut. 101/254 cases had clinically positive neck (cN+) out of which 89 cases showed actual metastasis to cervical lymph nodes. Out of the remaining 153 cases with clinically negative neck (cN0), 56 (36.6%) patients had occult lymph node metastasis, that is despite of having cN0, their histo-pathological specimens of neck dissections showed metastatic deposits of tumor in one or more lymph nodes (pN+). Thus a total of 145/254 patients had pN+ (Table 1).

Out of the 145 patients, 78 (53.79%) patients had loco regional metastasis to cervical lymph nodes level IB and /or IA. There were 31 patients having metastasis to lymph node levels II and/or II, while 27 of them showed metastatic deposits in lymph node levels III, with or without involvement of levels I and II. In the remaining 9 cases there were metastasis seen in lymph node levels IV and V with or without involvement of lymph node levels I, II and III (Table 2).

**Figure 1:**
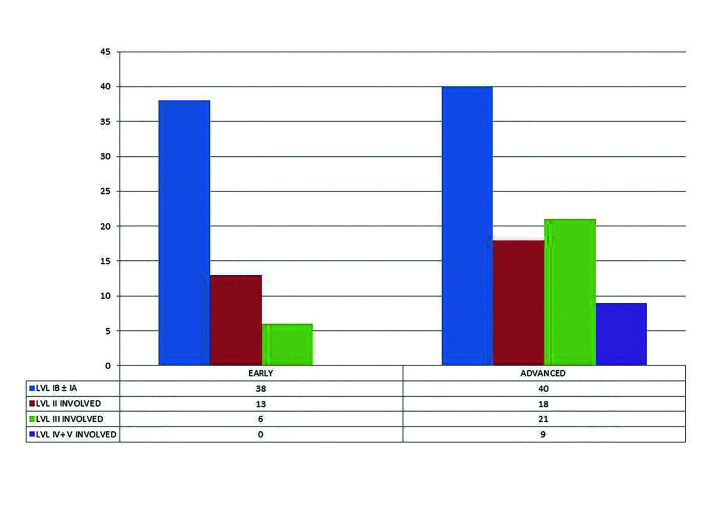
Relation between Tumor size and pattern of involvement of cervical lymph nodes (*p*<0.05).

When patients with pN+ were correlated with tumor size, it was seen that there was a statistically significant correlation between tumor size and lymph node metastasis (*p*<0.05). Out of 145 cases of pN+, 57 patients had an early tumor (<4cm) while 88 patients had an advanced tumor (>4cm). 38/57 patients with early tumor had level IB and/or IA lymph node involvement, 13/57 patients had level II and/or I lymph node involvement, 6/57 patients had level III involved, with or without involvement of level I and II lymph nodes, while there were no cases with early tumor which showed metastasis to level IV and V lymph nodes. When advanced cases were analyzed, it was seen that 40/88 had metastasis to level IB and/or IA lymph node, 18/88 had level II and/or level I lymph node involvement, 21/88 had level III involved, with or without involvement of level I and II lymph nodes, and 9/88 patients showed metastasis to level IV and V with or without involvement of level I, II or III lymph node (Fig. 1).

**Table 1:**
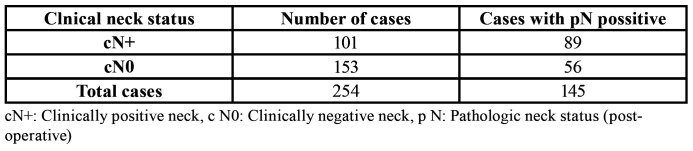
Clinical neck status of patients and their histo- pathological neck status.

**Table 2:**
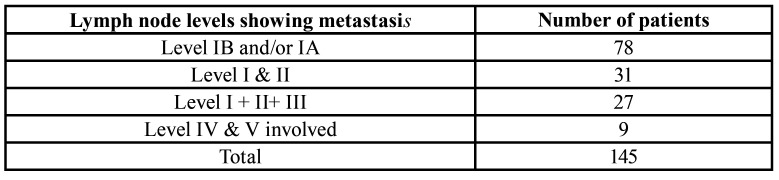
Detailed analysis of metastatic involvement of various lymph node levels.

While studying the relation between histo-pathological grading of tumor and cervical lymph node metastasis, Pearson correlation test showed that with reduction of degree of differentiation (as we go from well differentiated to poorly differentiated) the chances of metastasis to lymph nodes increases, and that was statistically significant (*p*<0.05). When association between histo-pathological grading and metastasis to individual lymph node levels were tested, the results came as statistically insignificant (*p*>0.05) (Table 3). Nonetheless, detailed analysis showed that for patients with well differentiated squamous cell carcinoma, out of 34 patients with pN+, 26 had metastasis to level IB and/or IA lymph nodes, 6 had metastasis to level II and/or I lymph node, 2 had metastasis to level III, with or without involvement of level I and II lymph nodes, while none of these patients had any metastasis to level IV and V lymph nodes.

**Table 3:**
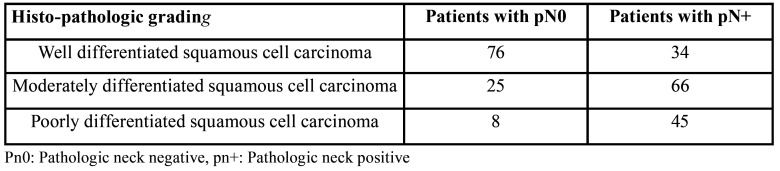
Histo-pathologic grading vs pathologic neck status.

**Figure 2:**
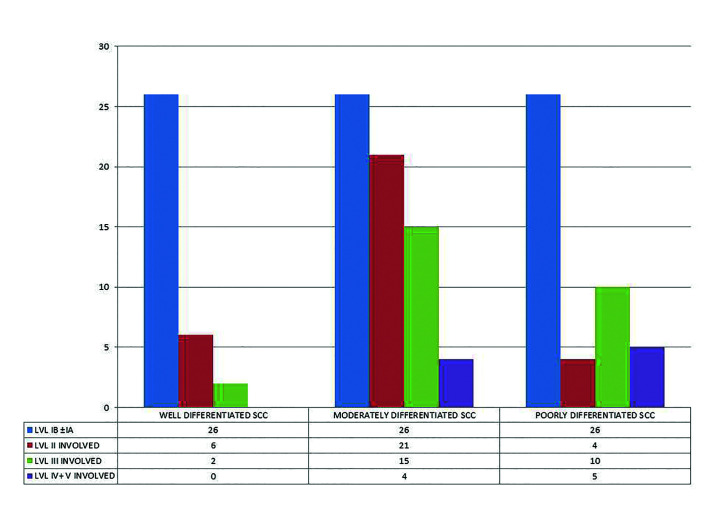
Relation between Histo-pathological grading and pattern of involvement of cervical lymph nodes (*p*>0.05).

In patients with moderately differentiated squamous cell carcinoma, out of 66 pN+ patients, 26 had metastasis to level IB and or IA lymph nodes, 21 had metastasis to level II and/or I lymph node, 15 had metastasis to level III, with or without involvement of level I and II lymph nodes and 4 had metastasis to level IV and V lymph nodes with or without the involvement of level I, II or III lymph nodes. For patients with poorly differentiated squamous cell carcinoma, out of 45 patients with pN+, 26 had metastasis in level IB and/or IA lymph nodes, 4 had metastasis to level II and/or I lymph node, 10 had metastasis to level III, with or without involvement of level I and II lymph nodes while 5 had metastasis to level IV and V lymph nodes with or without the involvement of level I, II or III lymph nodes (Fig. 2).

## Discussion

Loco-regional metastasis of OSCC to cervical lymph nodes is a very important prognostic factor. The number and size of affected lymph nodes, extra capsular spread and the extent of involvement of the neck, all contribute in determining the prognosis of a patient with oral carcinoma (8). Staging of patients is done also to define the need for adjuvant therapy in OSCC cases after surgery, because it is proven that in patients with pN+ post-operative radiotherapy reduces chances of recurrence (9,10).

The treatment of neck in patients with OSCC has seen several debates and controversies over the past years with the surgical treatment of the neck emerging as the best treatment regime (5,11). Thus it becomes very essential to map the locoregional metastasis of OSCC, so that we can understand the involvement of individual nodal levels better; which in turn will facilitate us to decide on the extent of neck dissections.

It is a proven fact that the metastasis to cervical lymph nodes varies from region to region with the tongue and floor of the mouth having the higher prevalence. Thus we decided to study loco-regional metastasis of cancer of a single site in the oral cavity (buccal mucosa) to overcome this bias (12,13). In India, majority of patients with OSCC report with lesion of the buccal mucosa and so OSCC of this region was chosen to study the pattern of cervical lymph node metastasis (14). Previously done studies have proven the fact that squamous cell carcinomas of the oral cavity along with oral sub mucous fibrosis (OSMF) should be considered a different entity as they are much less aggressive have comparatively lesser T and N stages; thus we decided not to include patients with OSMF in our study (15).

Contrast enhanced CT Scans (less than 1 month old) were chosen as the method to determine tumor size because of its high specificity and sensitivity (16). The tumor size mentioned in the resected specimens were not used in this study keeping in mind the shrinkage the specimen undergoes while being stored in 10% formalin. Various studies give various magnitude of shrinkage (11.3% to 14.9%) which might compromise the accurate recording of the size of the primary (17,18).

In our study, majority of the patients were females (149/254) which is in concordance with previous studies conducted on cancer patients from Indian population. This trend can be attributed to the more prevalence of habits in females like chewing betel nuts, tobacco or keeping a beetle leaf quid in mouth (14,19,20).

Out of 145 pN+ patients, 53.79% (78/145) had metastasis involving level IB and/or IA lymph nodes while 93.79% (136/145) had metastasis restricted to level I, II and III lymph nodes which is in Concorde to the study by J. P. Shah (12), though the number of cases where lymph node metastasis was confirmed histologically in this study was 57.09% (145/254), much lesser than the afore mentioned study. One of the probable reasons for this might be that our study dealt exclusively with carcinomas of buccal mucosa which has comparatively much lesser tendency to have cervical lymph node metastasis than carcinomas of tongue and floor of mouth (13). Out of 254 cases analysed, 101 patients had clinically positive neck out of which, 89 were proved to have metastatic spread histo-pathologically. For the rest 12 patients the lymph node enlargement was purely reactive. The prevalence of occult metastasis in our patient cohort was 36.6% (56/153) which was similar to the estimated rate predicted by similar studies in the past (21,22). Only 9 out of 145 pN+ cases (6.2%) showed metastasis to level IV and V lymph nodes, all which were cN+ and had advanced primary tumor (T>4cm) and moderately or poorly differentiated squamous cell carcinoma. 8 out of 9 cases were associated with multiple lymph node involvement of lymph node levels other than level IV and V while there was 1 case which showed only metastasis to level V lymph node. This is might be because of skip metastasis which is seldom seen in cases of buccal mucosa and more prevalent amongst tongue carcinoma cases.

There are various opinions regarding the effect of tumor size on cervical lymph node metastasis with few studies finding a significant correlation between the two while others failed (23,24). In our study though, we found that advanced lesions (T>4cm) had a higher propensity for lymph node metastasis. Amongst early cases with pN+, in 66.67% (38/57) patients the metastasis was only restricted to level IB and /or level IA lymph nodes, 22.81% (13/57) patients had metastasis in level I and II lymph nodes while only 10.52% (6/57) patients had metastasis involving level I, II and III lymph nodes. On the contrary amongst advanced cases, the patients with metastasis to level IB and/or IA dropped to 45.45% (40/88) while 10.22% (9/88) cases showed metastasis to level IV and V lymph nodes. In 20.45% of cases metastasis was seen involving lymph node levels I and II and in 23.86% of cases lymph node levels I, II and III were involved. While analysing histo-pathological grading with cervical lymph node metastasis, we noted that well differentiated SCC had the least occurrences of cervical lymph node metastasis while the poorly differentiated variety had the most. Well differentiated SCC is said to maintain their cellular cohesiveness and expresses higher adhesion molecules (like E-cadherin) than poorly differentiated SCC. Thus poorly differentiated carcinomas show a more aggressive pattern at the tumor-host interface, which might be responsible for higher chances of metastasis (25). Although no statistical significance was seen between histo-pathologic grading and the pattern of involvement of various lymph node levels, micro-analysis revealed that out of all pN+ cases with well differentiated squamous cell carcinoma, 95.59% (26/34) had metastasis to only level IB and/or IA lymph nodes. Amongst the patients with moderately differentiated squamous cell carcinoma, 39.39% (26/66) showed metastasis to level IB and/or IA, 31.82% (21/66) showed metastasis to level I and II lymph nodes while 22.73% (15/66) showed involvement of level III lymph nodes with or without involvement of level I and II lymph nodes. The patients with poorly differentiated squamous cell carcinoma showed a higher prevalence of involvement of level IV and V lymph nodes (11.09%).

This study has it draw backs in being a retrospective and single centre study. Due to the retrospective nature of the study, we had no data about the presence of Human Papilloma Virus (HPV) in any of our patients. In view of the association of HPV with OSCC, it should be worthwhile to conduct further experimental studies to elucidate its role in oral carcinogenesis of buccal mucosa. However a detailed analysis of prevalence of metastasis at various levels of lymph nodes and its correlation with tumor size and histo-pathologic grading has helped us to infer that cases of advanced tumor size and poor histo-pathologic differentiation show more tendencies of involvement of level IV and V lymph nodes. Thus, in such cases an extensive removal of all five lymph node levels are advocated.

## Conclusions

To infer, this study showed that in majority of the patients having squamous cell carcinoma of the buccal mucosa, the main group of lymph nodes that are involved are level I, II and III lymph nodes; out of which level IB and/or IA is the most frequently involved node. Rarely metastasis to level IV and V lymph nodes could be appreciated, especially in patients with advanced primary tumor and poor histo-pathologic differentiation.
